# Employment Trends During Preschool Years Among Mothers of Term Singletons Born with Low Birth Weight

**DOI:** 10.1007/s10995-014-1468-1

**Published:** 2014-03-19

**Authors:** Lars Johan Hauge, Tom Kornstad, Ragnhild Bang Nes, Petter Kristensen, Lorentz M. Irgens, Markus A. Landolt, Leif T. Eskedal, Margarete E. Vollrath

**Affiliations:** 1 Division of Mental Health, Norwegian Institute of Public Health, Post Box 4404, Nydalen, 0403 Oslo, Norway; 2 Research Department, Statistics Norway, Oslo, Norway; 3 Department of Occupational Medicine and Epidemiology, National Institute of Occupational Health, Oslo, Norway; 4 Institute of Health and Society, University of Oslo, Oslo, Norway; 5 Medical Birth Registry of Norway, Norwegian Institute of Public Health, Bergen, Norway; 6 Department of Global Public Health and Primary Health Care, University of Bergen, Bergen, Norway; 7 Department of Psychosomatics and Psychiatry, University Children’s Hospital, Zurich, Switzerland; 8 Research Department, Sørlandet Hospital, Kristiansand, Norway; 9 Psychological Institute, University of Oslo, Oslo, Norway

**Keywords:** Birth weight, Child care, Employment, Special health care needs, Work participation

## Abstract

Children born at term with low birth weight (LBW) are regarded growth restricted and are at particular risk of adverse health outcomes requiring a high degree of parental participation in the day-to-day care. This study examined whether their increased risk of special health care needs compared to other children may influence mothers’ opportunities for participation in the labor market at different times after delivery. Data from 32,938 participants in the population-based Norwegian Mother and Child Cohort Study with singleton children born at term in 2004–2006 were linked to national registers in order to investigate the mothers’ employment status when their children were 1–3 years in 2007 and 4–6 years in 2010. Children weighing less than two standard deviations below the gender-specific mean were defined as LBW children. Although not significantly different from mothers of children in the normal weight range, mothers of LBW children had the overall highest level of non-employment when the children were 1–3 years. At child age 4–6 years on the other hand, LBW was associated with an increased risk of non-employment (RR 1.39: 95 % CI 1.11–1.75) also after adjustment for factors associated with employment in general. In accordance with employment trends in the general population, our findings show that while mothers of normal birth weight children re-enter the labor market as their children grow older, mothers of LBW children born at term participate to a lesser extent in paid employment and remain at levels similar to those of mothers with younger children.

## Introduction

Improved neonatal care has contributed to increased survival among preterm and low birth weight (LBW) infants [1, 2]. However, the risk for chronic conditions requiring a high degree of parental participation in the day-to-day care increases markedly with decreasing birth weight and gestational age [3–6]. While LBW children (i.e., <2.500 g) born preterm can be appropriate, constitutional, or pathological small for their gestational age (SGA), most LBW children born at term are SGA and many have failed to reach expected growth in utero and are regarded growth restricted [7, 8]. The risk of perinatal morbidity and mortality increases the longer the slow growth has occurred [9, 10], and growth restricted children are at particular risk of severe intellectual disabilities and cerebral palsy, even exceeding the risk found among children born preterm [11–13]. The severity and chronicity of many conditions prevalent in these children can have a profound effect on their health and functioning, and many are identified as having special health care needs (SHCN) [14–16]. These children’s care needs are often of a type and amount beyond that required by children of the same age in general, and can therefore, as primary caregivers, particularly impinge on mothers’ time budgets and opportunities for regular participation in the labor market [17–19].

Mothers of children with SHCN often report difficulties engaging in paid work, and more often than mothers in general to have missed days from work, to have cut work hours, or left paid employment altogether due to their child’s additional care needs [20, 21]. However, the association between child care and work participation is complex and dependent on factors related to the child (e.g., age), the mother herself (e.g., educational attainment), and the household at large (e.g., financial circumstances) [22]. Mothers in households that can manage on one salary may prefer to stay at home or to reduce work hours while their children are young, before re-entering or increasing their work participation sometime as their children reach preschool and school age [23, 24]. This typical pattern characterized by increasing work hours with increasing child age is not as apparent among mothers of children with SHCN [25].

Although LBW is associated with excess risk of morbidity and SHCN [4, 5], limited knowledge exists about how the care needs of LBW children born at term can influence their mothers’ opportunities for participation in the labor market. Through linkage of population-based data from national registers, this study aimed to investigate associations between children’s birth weight and mothers’ risk of non-employment during preschool years. In order to examine employment trends as children age, we investigated mothers’ employment status when the children were 1–3 years, and again when the children were 4–6 years of age. As the risk of non-employment has been found to be most pronounced among mothers of children with severe care needs [21, 22], we expect the risk to be increasing with decreasing birth weight. Moreover, due to the adverse health outcomes observed among many LBW children and as women in general increase their work participation as children age [24], we expect the association between the child’s birth weight and maternal work participation to become more pronounced as the child grows older.

## Methods

### Participants, Linking Procedure and Inclusion Criteria

The study population comprised participants in the population-based Norwegian Mother and Child Cohort Study (MoBa), conducted by the Norwegian Institute of Public Health [26]. The study recruited pregnant women at their first routine ultrasound examination around weeks 17–18 of gestation from 1999 to 2008, and includes about 107,000 pregnancies in total. The MoBa cohort is linked to the Medical Birth Registry of Norway, which is based on compulsory notification of all deliveries in Norway with more than 12 completed weeks of gestation [27]. The registry contains the national identification number for all participants in the study, allowing linkage with the Central Population Register, the National Education Database of Statistics Norway, and the employment register in the National Insurance Administration. This linkage provided longitudinal data with annual updates for both mothers and children through 2010.

For the purpose of this study, mothers with children born in 2004–2006 were selected, of whom 43.5 % of those invited consented to participate in the MoBa study [28]. Since several thousand women have participated with more than one pregnancy (>15 %), and as participation in the study was linked to national registers, only unique pairs of mothers and children were eligible. Altogether, data for 40,502 women were successfully linked to the registries. Among these cases, twin and triplet births (1.7 %) and deliveries prior to week 37 and after week 41 of gestation, were excluded (12.9 %), leaving 34,587 cases eligible for the study. Among the eligible cases, we excluded cases where the mother had emigrated or where either the mother or the child had died (1.9 %), children born with severe congenital malformations (2.6 %), and cases for which data on birth weight or gestational age were missing (0.3 %), leaving a sample of 32,938 mothers and children who were residents of Norway in 2010. The study was approved by the Regional Committee for Medical Research Ethics in south-eastern Norway.

### Measures

The study outcome was maternal non-employment in 2007 and 2010. Employment status is reported annually by employers and recorded in the employment register in the National Insurance Administration. As the total labor force constitutes the sum of all employed and unemployed persons, non-employment differs from the concept of unemployment. As conceptualized in this study, non-employment is therefore defined as both the unemployed and those outside the labor force due to other reasons than unemployment. The proportion of women in the labor force of comparable age as the study participants’ age in 2007 (*M* 31.9: *SD* 4.7) was in the range of 82–85 % in 2007, and 81–86 % 3 years later in 2010. At the same time, the general unemployment rates among women were low, with 2.5 and 3.0 % unemployment in 2007 and 2010, respectively [29, 30].

The Medical Birth Registry of Norway provided data on birth weight, gestational age, year of birth, child’s sex, parity, and mother’s age and marital status at childbirth. Birth weight was standardized for gender into z-scores with a mean birth weight of zero applying data from all singleton live births in the total MoBa cohort. Mean birth weight was 3,535 g (*SD* 548 g) for girls and 3,660 g (*SD* 573 g) for boys. A one unit decrease in the z-score equals a decrease in birth weight of one standard deviation, and children weighing less than two standard deviations below the mean were defined as LBW children (boys < 2.510 g: girls < 2.436 g), all of whom were SGA below the 10th percentile according to Norwegian birth weight by gestational age standards [8]. Children with birth weights ranging from the mean to one standard deviation above the mean were selected as referents.

Data on educational attainment was obtained from the National Education Database of Statistics Norway according to a six-digit code reflecting nine discrete levels of education [31]. The women’s highest level of education at childbirth was categorized as educational attainment below high school graduate (0–3), high school graduate (4–5), lower college or university level (6), and higher college or university level, including postgraduate levels (7–8).

An indicator of the household’s dependency on the mother’s income was obtained from questionnaire assessments provided by the participants in the MoBa study about week 17 of gestation. The women were asked *Is it possible for your household to manage financially without your income*?, and we considered responses from women reporting *without difficulties* as households not dependent on the mothers’ income.

### Statistical Analysis

As there is considerable variation in work participation among mothers of toddlers and preschool children [24], we first investigated the mothers’ risk of non-employment in 2007 when their children were 1–3 years, and again in 2010 when their children were 4–6 years of age, adjusted for several factors associated with maternal employment in general. The child’s age was constructed as the difference between the year of follow-up and the child’s birth year. Analyses were performed using Stata/SE version 12.1 [32] and as non-employment is common among women with small children, robust Poisson regression analyses were performed [33]. Results are presented as adjusted risk ratios (RR) with corresponding 95 % confidence intervals (CI). Apart from the child’s birth weight, the models included gestational age coded according to conventional cut-offs as early term (37–38 weeks) and full term (39–41 weeks) births [34], mother’s age, educational attainment, parity, and household type, all according to characteristics at childbirth, in addition to self-reported household dependency on own income and the child’s age at follow up in 2007 and 2010, respectively.

## Results

A total of 22.8 % of the mothers were not employed when their children were 1–3 years in 2007, dropping to 15.6 % when their children were 4–6 years in 2010. Mothers of the youngest children were less likely to be employed during early motherhood, while the child’s age influenced maternal employment status less when the children were 4–6 years. A trend towards lower work participation with decreasing child birth weight was evident, and the proportion of mothers not in paid employment was considerably higher across the whole birth weight distribution when the children were 1–3 years as compared to when the children were 4–6 years (Table 1). Although mothers of LBW children had the overall highest level of non-employment when their children were 1–3 years (25.3 %), LBW was not independently associated with an increased risk of non-employment during early motherhood. Non-employment was common also among mothers of children with birth weights above the mean during this period (22.0 %), and factors such as low educational attainment, young age, and single-motherhood were more important for maternal employment status when the children were 1–3 years. However, when the children were 4–6 years, LBW was significantly associated with the mothers’ employment status (Crude RR 1.65: 95 % CI 1.31–2.08). LBW had a substantial independent effect on maternal employment status also after adjusting for other important factors such as educational attainment, single-motherhood, and non-dependence on the mother’s income (RR 1.39: 95 % CI 1.11–1.75). Among mothers of LBW children, 24.0 % were not employed when their children were 4–6 years as compared to 14.6 % among mothers of children with birth weights ranging from the mean to one standard deviation above the mean. As compared to their own employment levels when the children were 1–3 years, mothers of LBW children remained at a high level of non-employment with a relative decrease of only 1.3 % points during this period, in contrast to the referents that had a relative decrease of 7.4 % points. In fact, their level of non-employment at child age 4–6 years remained quite similar to that of mothers of young children aged 1–3 in the normal weight range.

**Table 1:** Adjusted risk ratios for non-employment at two different times after delivery among mothers of children born at term in Norway during 2004–2006

	Number	Child’s age 1–3 years (2007)			Child’s age 4–6 years (2010)		
		% Not employed	RR	95 % CI	% Not employed	RR	95 % CI
Total	32,938	22.8			15.6		
*Birth weight*							
>1.00 SD above mean	4,134	22.2	1.01	0.95–1.08	14.5	0.99	0.91–1.07
0.00–0.99 SD above mean	12,660	22.0	1.00	Reference	14.6	1.00	Reference
0.01–1.00 SD below mean	12,797	23.4	1.03	0.98–1.08	16.5	1.04	0.95–1.14
1.01–2.00 SD below mean	3,114	23.7	1.01	0.95–1.09	16.7	1.09	1.02–1.14
<2.00 below mean	233	25.3	1.01	0.81–1.27	24.0	1.39	1.11–1.75
*Gestational age*							
Full term (weeks 39–41)	26,829	22.7	1.00	Reference	15.3	1.00	Reference
Early term (weeks 37–38)	6,109	23.0	0.97	0.93–1.03	17.0	1.04	0.97–1.10
*Mother’s age*							
24 years and younger	4,032	37.9	1.54	1.45–1.63	25.2	1.31	1.21–1.41
25–29 years	11,068	21.6	1.13	1.07–1.18	14.8	1.10	1.04–1.17
30 years and older	17,838	20.1	1.00	Reference	13.8	1.00	Reference
*Educational attainment*							
<High school graduate	2,800	45.1	2.02	1.87–2.17	36.2	2.85	2.59–3.14
High school graduate	7,444	27.6	1.33	1.24–1.43	20.8	1.76	1.61–1.93
Lower college/university	17,332	18.3	0.96	0.90–1.02	11.4	1.02	0.93–1.11
Higher college/university	5,362	18.9	1.00	Reference	11.1	1.00	Reference
*Parity*							
1st	16,333	23.8	1.00	Reference	15.7	1.00	Reference
2nd	10,492	19.7	0.94	0.90–1.00	14.1	0.97	0.91–1.03
3rd	4,797	23.5	1.13	1.06–1.20	16.3	1.09	1.00–1.18
4th or higher	1,316	32.2	1.41	1.29–1.54	23.0	1.34	1.20–1.49
*Household type*							
Two-parent	31,634	21.9	1.00	Reference	14.9	1.00	Reference
Single-mother	1,304	45.2	1.55	1.45–1.66	32.0	1.56	1.44–1.70
*Dependency on mother’s income*							
Household dependent	27,481	21.5	1.00	Reference	14.8	1.00	Reference
Household not dependent	5,457	29.4	1.41	1.35–1.48	19.5	1.38	1.30–1.46
*Child’s age in 2007 and 2010*							
1/4 years	11,379	26.8	1.35	1.29–1.42	16.2	1.12	1.06–1.19
2/5 years	10,683	20.8	1.03	0.98–1.08	15.2	1.02	0.96–1.08
3/6 years	10,876	20.5	1.00	Reference	15.2	1.00	Reference

All categories according to characteristics at childbirth, except child’s age which represents age at follow-up in 2007 and 2010

## Discussion

This study shows that there is a lasting association between children’s birth weight and mothers’ employment status during preschool years, and that the risk of non-employment generally increases with decreasing birth weight. The excess morbidity and elevated care needs observed in LBW children born at term appear to influence mothers’ opportunities for participation in the labor market for an extended period of time. We observed a negative association between birth weight and work participation becoming more pronounced as the child grows older, also after adjustment for important factors associated with employment in general. Although a somewhat smaller proportion of mothers of LBW children were out of paid employment as their children age, the employment levels among mothers of children in the normal weight range increased markedly during this period, corresponding well to employment trends in the general Norwegian population [24]. Mothers of LBW children, on the other hand, remained at levels similar to those of mothers with young children in the normal weight range. The association observed between birth weight and work participation likely reflects a higher rate of health problems and disability in LBW children born at term, indicating that many children in the lower weight range require parental care and supervision above that needed by children of comparable age within the normal weight range. Our findings thus correspond with studies showing elevated levels of SHCN among children in the lower weight range [4–6], and mirror studies showing a trend towards lowered maternal work participation in accordance with the severity of the child’s condition [21, 22].

To our knowledge, this is the first study to investigate associations between children’s birth weight and mothers’ employment status. Although a number of studies have investigated associations between children’s SHCN and mothers’ work participation, previous studies have almost exclusively utilized cross-sectional study designs, been based on parental report, or lacked appropriate reference groups of children without SHCN [25, 35]. Using a large population-based sample and register-based data to longitudinally investigate the risk of non-employment thus constitutes a major strength. The observed employment trends suggest that birth weight constitutes a valid indicator of children’s care needs in early life in which other objective data of children’s SHCN can be hard to obtain. Because the impact of different conditions varies accordingly, a child’s care needs must be considered in the context of the child’s stage of development [15]. Thus, in order to examine the relative effect of caring for a child with special needs, it is important to contrast the work participation of mothers of children with SHCN with that of mothers in general with children of comparable age. For instance, our finding that children’s birth weight affects mothers’ work participation to a larger extent as children age contradicts conclusions drawn in studies utilizing samples solely of children with SHCN [21, 22]. Although mothers of LBW children had the overall highest level of non-employment when the children were 1–3 years, reduced work participation during early motherhood is common also in the general population [25]. Norwegian figures of female labor market participation show that approximately 1 in 4 women with small children are non-employed, dropping to 1 in 5 as children grow older [24]. With close to 90 % of children in kindergarten [30] and as Norwegian unemployment rates are low and well below the OECD average [36], most non-employed women are out of paid employment due to other reasons than lack of child care and unemployment. Thus, because many women are not employed for shorter and longer periods when their children are young, elevated levels of non-employment among mothers of children with SHCN need not solely reflect the additional care needs of these children, but rather a general trend towards lowered work participation during early motherhood. As we have shown, other important factors such as educational attainment and household income greatly influence on women’s employment choices, irrespective of their children’s care needs.

Some limitations of the present study need to be recognized. Although a consistent trend towards higher risk of non-employment with decreasing birth weight is evident, some caution is warranted due to a relative small number of LBW children. The response rate in MoBa is also lower than optimal, and although not uncommon for large epidemiological studies [37], self-selection to the study may result in deviations from the larger population from which the women were sampled. Comparisons of cohort participants with all women giving birth in Norway during the same period identified several deviations in prevalence estimates [28], and of particular interest to the present study is the under-representation of single-mothers, women below 25 years of age, and that infants in MoBa had a somewhat higher mean birth weight and were on average born after longer gestation. In terms of birth weight and gestational age, the relative differences were small. The fact that the youngest women are less represented, and as young women has the overall lowest employment rates [29, 30], may imply a bias towards higher work participation in this sample as compared to the general population. As somewhat older women tend to have completed their education, are more likely to be stable employed, and thus to experience a more favorable economic situation, they may also have better opportunity to temporarily stay at home or to reduce work hours. Nevertheless, the overall employment rates in the present sample correspond fairly well with that of women with young children in the general Norwegian population [24]. Moreover, apart from information reflecting single-mother versus two-parent households, the study lacks objective information on paternal factors such as educational attainment and employment status. However, although the total household income is dependent on such factors, and further, that household income is likely to impact on the employment choices of women with small children [23], the subjective assessment regarding dependency on own income used in this study may better reflect the individual realities as they are perceived by the members of the household as a whole. Notwithstanding the limitations above, as informed consent to participate in the study was obtained during early pregnancy, prior to any knowledge of pregnancy outcomes or children’s health care needs, and as the register-based data ensures complete follow-up of all participants who were residents of Norway in 2010, the present study constitutes a powerful alternative to traditional longitudinal approaches which often suffer from a large loss to follow-up and systematic attrition [38].

## Conclusion

In accordance with other studies on employment among mothers of children with SHCN, it appears that mothers of LBW children do modify their employment behavior in order to meet the health care needs of their children. Their higher risk of non-employment may result in these women becoming increasingly disadvantaged with regard to future career prospects, earnings potential, and pension benefits. Although our sample comprises only young children, many of the conditions prevalent in LBW children persist throughout adolescence and young adulthood, while other conditions have a later onset and are discovered only as the child grows older [39, 40]. As LBW children are at higher risk of future academic under-achievement [41], unemployment [42], and being granted early disability pension [43], this implies that the health problems and care needs of many of these children persist for an extended period of time in which other healthy children gradually become more independent. Their care needs are thus likely to inhibit future maternal employment prospects also as the children grow older. Disrupted occupational careers may prevent many women from utilizing the beneficial effects of stable employment, possibly resulting in poorer caregiver health that in turn can have detrimental effects for the welfare of the entire family in the long run [44, 45]. In order to reduce long-term negative consequences of non-employment among those who have a preference to participate in the labor market, it will be important to initiate arrangements for these mothers that make it possible to remain employed while at the same time meeting the care needs of their children.

## Abbreviations

LBW: Low birth weight
MoBa: The Norwegian Mother and Child Cohort Study
SGA: Small for gestational age
SHCN: Special health care needs
